# NADPH Oxidase 5 and Melatonin: Involvement in Ram Sperm Capacitation

**DOI:** 10.3389/fcell.2021.655794

**Published:** 2021-05-07

**Authors:** Sara Miguel-Jiménez, Blanca Pina-Beltrán, Silvia Gimeno-Martos, Melissa Carvajal-Serna, Adriana Casao, Rosaura Pérez-Pe

**Affiliations:** Grupo BIOFITER-Departamento de Bioquímica y Biología Molecular y Celular – Instituto Universitario de Investigación en Ciencias Ambientales de Aragón (IUCA), Facultad de Veterinaria, Universidad de Zaragoza, Zaragoza, Spain

**Keywords:** ram spermatozoa, capacitation, NADPH oxidase, NOX5, melatonin, reactive oxygen species

## Abstract

Reactive oxygen species (ROS) play an essential role in mammalian sperm capacitation. NADPH oxidase 5 (NOX5) has been described as the main source of ROS production in some mammalian spermatozoa, such as human and equine. On the other hand, melatonin can decrease cellular ROS levels and regulates NOX activity in somatic cells. Therefore, the objectives of this work were (1) to identify NOX5 in ram spermatozoa and analyze its possible changes during *in vitro* capacitation and (2) to investigate the effect of melatonin on NOX5 expression and localization and on superoxide levels in capacitated ram spermatozoa. Protein bands associated with NOX5 were detected by Western blot analysis. Likewise, indirect immunofluorescence (IIF) revealed six different immunotypes for NOX5, which varied throughout *in vitro* capacitation. Superoxide (O_2_^⋅–^), evaluated by DHE/Yo-Pro-1, rose after *in vitro* capacitation and in the presence of the calcium ionophore A23187 but decreased in the presence of the NOX inhibitor GKT136901. GKT also reduced the percentage of capacitated and acrosome-reacted spermatozoa that had increased during incubation in capacitating conditions. The presence of melatonin at micromolar concentrations avoided the increment in O_2_^⋅–^ and the changes in NOX5 immunotypes provoked by capacitation. In conclusion, NOX5 is present in ram spermatozoa and the changes in its distribution, associated with sperm capacitation, can be prevented by melatonin. To this extent, it could imply that melatonin exerts its antioxidant role, at least in part, by modulating NOX5 activity during ram sperm capacitation.

## Introduction

Reactive oxygen species (ROS), such as superoxide anion (O_2_^⋅–^), are involved in sperm functionality under physiological conditions. ROS are essential for processes like capacitation, hyperactivation, acrosome reaction, and sperm-oocyte fusion (Griveau and Le Lannou, 1994; de Lamirande and Gagnon, 1995; O’Flaherty, 2015), but an excess can be detrimental to the sperm function (reviewed by Dutta et al., 2019).

It is well known that sperm cells by themselves can generate the superoxide anion (Aitken and Clarkson, 1987; Alvarez et al., 1987) which spontaneously or enzymatically dismutates to hydrogen peroxide (H_2_O_2_) (Halliwell and Gutteridge, 1989). The enzymes responsible for this superoxide production are the NADPH oxidases (NOX) (Bedard and Krause, 2007; Maghzal et al., 2012) and, more specifically, the NOX5 in mammal spermatozoa (Ghanbari et al., 2018). This isoform has some characteristics that differentiate it from other NOX family members (reviewed by Touyz et al., 2019). NOX5 does not need the NADPH oxidase subunit for its activation, but it is entirely calcium-dependent, thanks to the calcium-binding sites on its N-terminal. There is a high-level expression of NOX5 mRNA in the human testis (Banfi et al., 2001), and the presence of the enzyme has also been described in human (Musset et al., 2012), canine (Setyawan et al., 2016), and equine (Sabeur and Ball, 2007) spermatozoa. The fact that the addition of a calcium ionophore (A23187) to spermatozoa induces ROS generation (Aitken et al., 1992; de Lamirande et al., 1998) suggests that NOX5 is the primary source of ROS in these cells (Banfi et al., 2001). However, there is no NOX5 gene or protein in murine spermatozoa, which indicates that, at least in this species, another source must be involved in the superoxide generation (Vernet et al., 2001), such as mitochondria (Koppers et al., 2008) or inflammatory leukocytes (Whittington et al., 1999).

An increase in the production of ROS [superoxide anion (O_2_^–^), hydrogen peroxide (H_2_O_2_), nitric oxide (NO⋅), and peroxynitrite (ONOO^–^)] is one of the essential events that occur during sperm capacitation (de Lamirande and Gagnon, 1995; Herrero et al., 2003; O’Flaherty et al., 2004b). According to Ghanbari et al. (2018), the NOX5 enzyme must be involved in this process, due to its capacity to produce O_2_^–^. Other authors also assumed that the superoxide produced by NOX5, and the peroxides derived from it, activate the adenylyl cyclase, and thus the cAMP-PKA pathway (Zhang and Zheng, 1996; O’Flaherty, 2015; Moreno-Irusta et al., 2020), which finally leads to the phosphorylation of the tyrosine residues associated with sperm capacitation, as described in human and rat (Lewis and Aitken, 2001; O’Flaherty et al., 2004a). This process may also occur in ram spermatozoa. However, to the best of our knowledge, the NOX5 isoform has not been identified in ram spermatozoa.

Ovine reproduction has a marked seasonality, controlled by the secretion of melatonin from the pineal gland. This hormone directly affects the synthesis and secretion of the gonadotropic hormones via the hypothalamic–pituitary axis (Bittman et al., 1983). It is also synthesized by the reproductive tract (Gonzalez-Arto et al., 2016), and it is present in reproductive fluids, such as seminal plasma (Casao et al., 2010a) or follicular fluid (Brzezinski et al., 1987). Thus, as we have demonstrated over recent years, melatonin directly affects ram sperm functionality and modulates capacitation, decreasing apoptosis markers and oxidative stress (Casao et al., 2010b; Gimeno-Martos et al., 2019; Miguel-Jimenez et al., 2020). The well-known antioxidant capacity of melatonin may be the reason for its anti-apoptotic action since elevated ROS levels lead to early cell death (Rao and Gangadharan, 2008; Casao et al., 2010b; du Plessis et al., 2010; Jang et al., 2010; Espino et al., 2011; Gimeno-Martos et al., 2019; Miguel-Jimenez et al., 2020). Furthermore, a recent study in somatic cells (Patino et al., 2016) found an antioxidant melatonin effect throughout NOX regulation.

Considering NOX5 as the principal source of ROS in mammalian spermatozoa, the involvement of ROS in sperm capacitation and the effects of melatonin on regulating this process in ram, we set out to determine whether the effect of this hormone on ram sperm capacitation is mediated by the reduction of superoxide levels through NOX5 activity modulation. For this purpose, the objectives of this work were to detect the NOX5 isoform in ram spermatozoa (1), to determine superoxide and NOX5 involvement in ram sperm capacitation (2), and finally, to investigate how melatonin affects the expression and localization of NOX5 and superoxide levels in capacitated ram spermatozoa (3).

## Materials and Methods

Unless otherwise stated, all reagents were purchased from Merck KGaA (Darmstadt, Germany).

### Semen Collection and Processing

Semen was collected from nine *Rasa Aragonesa* rams (2–4 years old) with the aid of an artificial vagina. All the rams belonged to the Rasa Aragonesa National Breeding Association (Asociación Nacional de Criadores de Ganado Ovino Selecto de Raza Rasa Aragonesa, ANGRA) and were kept at the Experimental Farm of the University of Zaragoza Veterinary School under the same nutritional conditions. All experimental procedures were performed under the project license PI39/17 approved by the University of Zaragoza Ethics Committee for Animal Experiments, following the Spanish Policy for Animal Protection RD53/2013, which meets the European Union Directive 2010/63 on the protection of animals used for experimental and other scientific purposes.

After 2 days of abstinence, two successive ejaculates were collected, and second ejaculates were pooled and processed together to avoid individual differences (Ollero et al., 1996). The ejaculates were kept at 37°C until analysis.

In order to obtain a plasma-free sperm population, a dextran/swim-up procedure (García-López et al., 1996) was performed in a medium with the following composition: 200 mM sucrose, 50 mM NaCl, 2.7 mM CaCl_2_, 18.6 mM sodium lactate, 21 mM HEPES, 10 mM KCl, 2.8 mM glucose, 0.4 mM MgSO_4_, 0.3 mM sodium pyruvate, 0.3 mM K_2_HPO_4_, 5 mg/mL bovine serum albumin (BSA), 30 mg/mL dextran, 1.5 IU/mL penicillin, and 1.5 mg/mL streptomycin, pH 6.5.

Sperm concentration was calculated in duplicate using a Neubauer chamber (Marienfeld, Lauda-Königshofen, Germany).

### *In vitro* Capacitation

Swim-up-selected spermatozoa, in aliquots of 1.6 × 10^8^ cells/mL, were incubated for 3 h at 39°C in a humidified incubator with 5% CO_2_ in the air. Incubations were performed in a complete TALP medium (Parrish et al., 1988) containing 100 mM NaCl, 3.1 mM KCl, 25 mM NaHCO_3_, 0.3 mM NaH_2_PO_4_, 21.6 mM Na lactate, 3 mM CaCl_2_, 0.4 mM MgCl_2_, 10 mM HEPES, 1 mM Na pyruvate, 5 mM glucose, and 5 mg/mL bovine serum albumin (BSA), with a pH of 7.3 (TALP samples). Several cAMP-elevating agents, already proven for capacitating ram spermatozoa (Grasa et al., 2006; Colas et al., 2008) and composed of 1 mM dibutyryl (db)-cAMP, 1 mM caffeine, 1 mM theophylline, 0.2 mM okadaic acid, and 2.5 mM methyl-b-cyclodextrin, were added to the TALP medium to induce *in vitro* capacitation (capacitated-control samples, Cap-C).

To further investigate the role of NOX5 during *in vitro* capacitation, the NOX inhibitor 2-(2-chlorophenyl)-4-methyl-5-(pyridin-2-ylmethyl)-1*H*-pyrazolo[4,3-*c*]pyridine-3,6(2H,5H)-dione (GKT136901, GKT) was added to the capacitation medium at final concentrations of 1 μM (Musset et al., 2012; Altenhofer et al., 2015). Taking into account that the activation of NOX5 is calcium-dependent (Banfi et al., 2001), 1 μM calcium ionophore A23187 was added to the sperm samples to evaluate the effect of NOX5 activation on changes related to capacitation.

Melatonin was solubilized in dimethyl sulfoxide (DMSO) and phosphate-buffered saline (PBS: 137 mM NaCl, 2.7 mM KCl, 8.1 mM Na_2_HPO_4_, and 1.76 KH_2_PO_4_, pH 7.4) and added to the capacitation medium at a final concentration of 1 μM. The final concentration of DMSO in all the melatonin samples was 0.1%. To account for the potential adverse effect of DMSO, the same concentration was included in capacitated-control samples to which no melatonin had been added.

Thus, the experimental groups in the present study were swim-up (spermatozoa selected by the dextran/swim-up method before inducing *in vitro* capacitation), TALP samples (spermatozoa incubated under capacitating conditions without cAMP-elevating agents), and capacitated-control (Cap-C, spermatozoa incubated under capacitating conditions in TALP medium with cAMP-elevating agents). Each compound (GKT136901, calcium ionophore A23187, melatonin or a combination of them) was added at 1 μM concentration to samples incubated in TALP and high-cAMP medium samples.

### Sperm Motility Evaluation

Total and progressive motility was evaluated using the motility module of OpenCASA, a free open-source software that we recently developed (Alquezar-Baeta et al., 2019). Two drops of 2 μL of each sample, diluted to a final concentration of 3 × 10^7^ cells/mL, were placed in a pre-warmed Makler counting chamber (Sefi Medical Instruments, Haifa, Israel) and maintained at 37°C during all the analyses by a heated slide holder. Spermatozoa were recorded with a video camera (Basler acA1920; Basler Vision Components, Ahrensburg, Germany) mounted on a microscope (Nikon Eclipse 50i, Nikon Instruments Int, Tokyo, Japan) equipped with a 10 × negative-phase contrast lens.

Recorded videos were evaluated with the following settings: 60 frames per second, 120 frames, 800 × 600 pixel image resolution, 10 μm^2^ minimum cell size, 100 μm^2^ maximum cell size, STR (straightness coefficient) > 80% and VAP (mean velocity) > 90%, 10 μm/s minimum VCL (curvilinear velocity), 100 μm/s VCL lower threshold, 200 m/s VCL upper threshold, 30 frames minimum track length, and 20 μm maximum displacement between frames.

### Flow Cytometry Analysis

All the measurements were performed on a Beckman Coulter FC 500 flow cytometer (Beckman Coulter Inc., Brea, CA, United States) equipped with two excitation lasers (air-cooled Argon ion laser 488 nm and Red Solid state laser 633 nm); five absorbance filters (FL1-525, FL2-575, FL3-610, FL4-675, and FL5-755; ± 5 nm each band pass filter); and CXP software. A minimum of 20,000 events were evaluated in all the experiments. The sperm population was identified for further analysis on the basis of its specific forward (FS) and side-scatter (SS) properties; thus, other events were excluded. A flow rate stabilized at 200–300 cells/s was used.

#### Sperm Membrane Integrity

Sperm viability, considered as the integrity of the cell plasma membrane, was assessed by adding 3 μL of 1 mM carboxyfluorescein diacetate (CFDA), 1.5 mM propidium iodide (PI), and 5 μL of formaldehyde (0.5% (v/v) in water) to a final concentration of 5 × 10^6^ cells/mL in a 300 μL volume, based on a modification of the procedure described by Harrison and Vickers (1990). Samples were incubated at 37°C in darkness for 15 min. For the sperm viability analysis, the Argon laser and filters FL1-525 and FL4-675 nm were used to avoid overlapping. The monitored parameters were FS log, SS log, FL1 log (CFDA), and FL4 log (PI). The percentage of viable spermatozoa (PI-/CFDA+) was evaluated.

#### Superoxide Levels

To detect intracellular superoxide (O_2_^⋅–^) levels in viable spermatozoa, cells (500 μL at 5 × 10^6^ cells/mL) were loaded with 4 μM DHE and 40 nM Yo-Pro-1 (Thermo Fisher Scientific, Waltham, MA, United States) fluorochromes (Guthrie and Welch, 2006) and incubated at 37°C for 40 min in darkness. Hydroethidine is permeable to cells, and it is oxidized by O_2_^⋅–^ to the red fluorescent compound ethidium (E), which was detected with the FL4-675-nm filter in the flow cytometer. Yo-Pro-1 labels cells with destabilized membrane (non-viable) with green fluorescence, detectable with the FL1-525-nm filter. Viable cells with intact membrane do not show fluorescence. Dot plots showed four different populations: viable cells with high O_2_^⋅–^ production (Yo-Pro-1-/E+), viable cells with low O_2_^⋅–^ production (Yo-Pro-1-/E-), non-viable cells with low O_2_^⋅–^ production (Yo-Pro-1+/E-), and non-viable cells with high O_2_^⋅–^ production (Yo-Pro-1+/E+). The percentage of viable spermatozoa with high superoxide levels (Yo-Pro-1-/E+) was evaluated.

#### Intracellular Calcium

To assess intracellular calcium levels, aliquots of 500 μL (5 × 10^6^ cells/mL) were incubated with 2 μM Fluo-4-AM and 0.02% pluronic acid for 15 min at 37°C in darkness (both from Thermo Fisher Scientific, Waltham, MA, United States) and 1.5 mM PI. After this, a flow cytometer was used to evaluate the stained samples, as described previously, using the Argon laser and filters FL1-525 and FL4-675 nm. The monitored parameters were FS log, SS log, FL1 log (Fluo-4-AM), and FL4 log (PI). The percentage of viable spermatozoa with high calcium levels (Fluo-4-AM+/PI-) was evaluated.

### Determination of Capacitation Status

The sperm capacitation state was evaluated using the chlortetracycline (CTC) assay (Ward and Storey, 1984) that we previously validated for the evaluation of capacitation and acrosome reaction-like changes in ram spermatozoa (Grasa et al., 2006). A CTC solution (750 μM; Sigma-Aldrich Corp., St. Louis, MO, United States) was prepared daily in a buffer containing 20 mM Tris, 130 mM NaCl, and 5 μM cysteine, pH 7.8, and passed through a 0.22-μm filter. After that, 20 μL of CTC solution was added to 18 μL of each sperm sample, fixed with 5 μL of 1.25% (w/v) paraformaldehyde in 0.5 M Tris–HCl (pH 7.8) and incubated at 4°C in the dark for 30 min. Six microliters of the stained sample was placed onto a glass slide and mixed with 2 μL of 0.22 M 1,4-diazabicyclo[2.2.2]octane (DABCO) in glycerol:PBS (9:1 v/v). The samples were covered with 24 × 60 mm coverslips, sealed with transparent enamel, and stored in the dark at −20°C until evaluation. The samples were examined using a Nikon Eclipse E-400 microscope (Nikon Corporation, Kanagawa, Japan) under epifluorescence illumination with a V-2A filter, and at least 200 spermatozoa were scored per sample. Three sperm patterns were identified (Gillan et al., 1997): non-capacitated (even distribution of fluorescence on the head, with or without a bright equatorial band), capacitated (with fluorescence in the acrosome), and acrosome-reacted cells (showing no fluorescence on the head, with or without a bright equatorial band).

### Indirect Immunofluorescence

NOX5 localization was revealed by indirect immunofluorescence analyses (IIF). Sperm samples were diluted (2 × 10^6^ cells/mL) in PBS and fixed in 0.5% (v/v) formaldehyde at room temperature for 20 min. Then, cells were centrifugated at 900 × *g*, and the pellet was resuspended in 500 μL PBS. Forty microliters of cell suspension was placed onto Superfrost slides (Superfrost Plus; Thermo Fisher Scientific, Waltham, MA, United States) and permeabilized with 0.5% (v/v) Triton X-100 in PBS for 15 min. Afterward, the cells were fixed again with paraformaldehyde 1.25% (w/v) in Tris–HCl 0.5 M for 5 min and washed three times with PBS. Non-specific binding sites were blocked with 5% (w/v) BSA in PBS for 5 h in a wet chamber. Slides were rewashed in PBS and incubated at 4°C overnight in a wet chamber with the primary antibody anti-NOX5 (Abcam, Cambridge, UK Cat# ab191010) 1/25 in PBS with 1% (v/v) BSA. The next morning, the samples were washed three times with PBS and incubated with the secondary antibody (Alexa Fluor 488 chicken anti-rabbit; Thermo Fisher Scientific; Cat#A-21441, RRID:AB_2535859), diluted 1/600 (v/v) in PBS with 1% (v/v) BSA for 90 min at room temperature in a wet chamber. Slides were then washed three times with PBS before the addition of 6 μL of 0.22 M 1,4-diazabicyclo[2.2.2]octane (DABCO) in glycerol:PBS (9:1 v/v) to enhance and preserve cell fluorescence. The slides were covered with a coverslip and sealed with transparent enamel. Cells were visualized with a Nikon Eclipse E400 microscope (Nikon, Tokyo, Japan) under epifluorescence illumination using a B-2A filter (×1,000). All samples were processed in duplicate, and at least 150 spermatozoa were scored per slide.

The specificity of the anti-NOX5 antibody was tested by the peptide blocking method. The antibody was neutralized with the immunizing blocking peptide (ProteoGenix, Schiltigheim, France) at a final concentration of 2.5 μg/mL in PBS with 1% BSA for 2 h at room temperature. The neutralized antibody was then used side by side with the antibody alone in two identical slides, and the results were comparatively analyzed. No signal was detected when using the neutralized antibody, which means that the antibody has high affinity for the NOX5 protein.

### Western Blotting

For NOX5 detection, sperm proteins were extracted from spermatozoa, as previously described by Colas et al. (2008). Aliquots of 3.2 × 10^7^ cells were centrifuged at 900 × g for 5 min. The supernatant was discarded, and the pellet was resuspended in 200 μL of extraction sample buffer [ESB; 68.6mM Tris–HCl (pH 6.8), 2% SDS (w/v)] with a 10% protease inhibitor cocktail. After incubation at 100°C in a sand bath for 5 min, the samples were centrifuged again at 7,500 × g for 5 min at 4°C. The supernatant was recovered, and β-mercaptoethanol, glycerol, and bromophenol blue (in 10% glycerol) were added to final concentrations of 5, 1, and 0.002% (v/v), respectively. Lysates were stored at -20°C.

Sperm extracted proteins (50 μL, 1–2 mg/mL) were separated in one dimension by 10% sodium dodecyl sulfate polyacrylamide gel electrophoresis (SDS-PAGE) (Laemmli, 1970) and transferred onto a PVDF membrane using a transfer unit (Trans-Blot Turbo Transfer System, Bio-Rad, Hercules, CA, United States). Non-specific sites on the PVDF membrane were blocked with 5% BSA (w/v) in PBS for 4 h. The proteins were immunodetected by incubating overnight at 4°C with a rabbit primary anti-NOX5 (Abcam, Cambridge, UK Cat# ab191010) diluted 1/400 in 0.1% (v/v) Tween-20 in PBS containing 1% (w/v) BSA. A mouse anti-tubulin antibody (Santa Cruz Biotechnology Cat# sc-8035, RRID:AB_628408) was used as a loading control (dilution 1/1,000). We used the commercial HeLa cell lysate (Abcam, Cambridge, United Kingdom), recommended explicitly by the antibody manufacturer, as a positive control. After extensive washing with 0.1% (v/v) Tween-20 PBS, membranes were incubated for 75 min at room temperature with the secondary antibodies donkey anti-rabbit (IRDye 680RD Donkey anti-Rabbit IgG antibody, LI-COR Biosciences Cat# 926-68073, RRID:AB_10954442) and donkey anti-mouse (RDye 800CW Donkey anti-Mouse IgG antibody, LI-COR Biosciences Cat# 926-32212, RRID:AB_621847) diluted 1/15,000 in 0.1% (v/v) Tween-20 PBS containing 1% (v/v) BSA. After extensive washing, the membranes were scanned using the Odyssey CLx Imaging System (LI-COR Biosciences, Lincoln, NE, United States).

The specificity of the anti-NOX5 antibody was tested by the peptide blocking method, as in the IIF assay. The neutralized antibody was used side by side with the antibody alone in two identical western blot membranes, and the results were comparatively analyzed. No signal was detected when using the neutralized antibody, which means that the antibody has high affinity for the NOX5 protein.

Western blot images were quantified using Odyssey Clx Infrared Imaging System software (Li-COR Biosciences, Lincoln, NE, United States) to determine the NOX5 protein bands’ relative intensity, normalized to the tubulin control.

### Statistical Analysis

Differences between the groups in motility, superoxide levels, viability, CTC staining, and NOX5 immunotypes were analyzed by means of the chi-square test. Differences in NOX5 expression profiles evaluated by western blot were analyzed by ANOVA followed by the Bonferroni *post hoc* test after evaluation of the data distribution by the Kolmogorov–Smirnov test. All statistical analyses were performed using GraphPad Prism 5 (v. 5.03; GraphPad Software, La Jolla, CA, United States).

## Results

All data obtained from the sperm samples capacitated without cAMP-elevating agents (TALP samples) are shown in the Supplementary Material, as this treatment had scarce effects on spermatozoa. The most evident changes were obtained after capacitating with cAMP-elevating agents, so that we focused on them.

### NOX5 Immunodetection and Immunolocalization in Ram Spermatozoa

**FIGURE 1:**
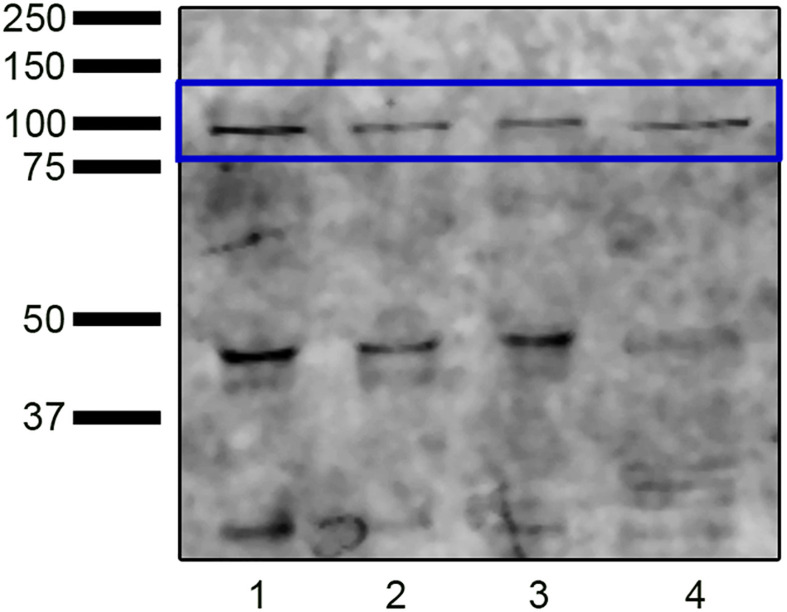
Western blot analysis revealing the presence of NADPH oxidase 5 in ram sperm protein extracts from swim-up selected samples (lane 1) and samples incubated in capacitating conditions without (TALP, lane 2) or with cAMP elevating agents (Cap-C, lane 3). Positive control (lane 4): HeLa cell lysate.

Western blot analysis identified protein bands with a molecular weight compatible with NOX5 (∼85 kDa) in swim-up, TALP samples, and capacitated control (Cap-C) samples (Figure 1). The molecular weights of these bands matched those found in the positive control. Besides, bands at 45 kDa and 30 kDa were detected.

**FIGURE 2:**
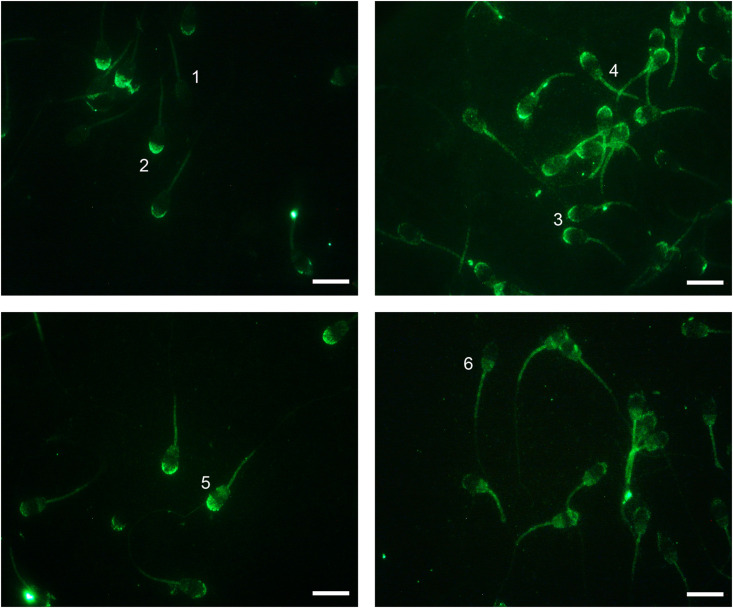
Indirect immunofluorescence (IIF) localization of NOX5 evaluated by fluorescence microscopy in ram spermatozoa. Six immunotypes can be seen: labeling in midpiece (1); in the acrosomal region (2); in the apical edge (3); in the apical edge + postacrosomal region (4); in the acrosomal and postacrosomal regions (5); and in the postacrosomal region (6). All of them present midpiece labeling. Original magnification ×1,000. Scale bars = 10 μm.

Immunofluorescence analysis revealed the presence of the NOX5 enzyme in ram spermatozoa, and six different immunotypes were distinguished (Figure 2): immunotype 1, with labeling only at the midpiece; immunotype 2, with labeled acrosomal region; immunotype 3, labeling the apical edge; immunotype 4, labeling the apical edge and postacrosomal region; immunotype 5, labeling acrosomal and postacrosomal regions; and immunotype 6, with postacrosomal labeling. Also, all the immunotypes presented fluorescence at the midpiece.

### Influence of NOX5 in Ram Sperm Capacitation

After demonstrating the presence of NOX5 in ram spermatozoa, we wanted to evaluate how the inhibition (by addition of GKT136901) or activation of NOX5 (by the increase in intracellular calcium provoked by the addition of calcium ionophore A23187) influenced some events related with sperm capacitation, such as changes in motility, CTC staining patterns, and superoxide levels. However, first, we verified that the addition of the ionophore was indeed capable of causing the calcium to enter inside the cell (Supplementary Figure 1).

#### Influence of NOX5 on Superoxide Intracellular Levels During Incubation in Capacitating Conditions

**FIGURE 3:**
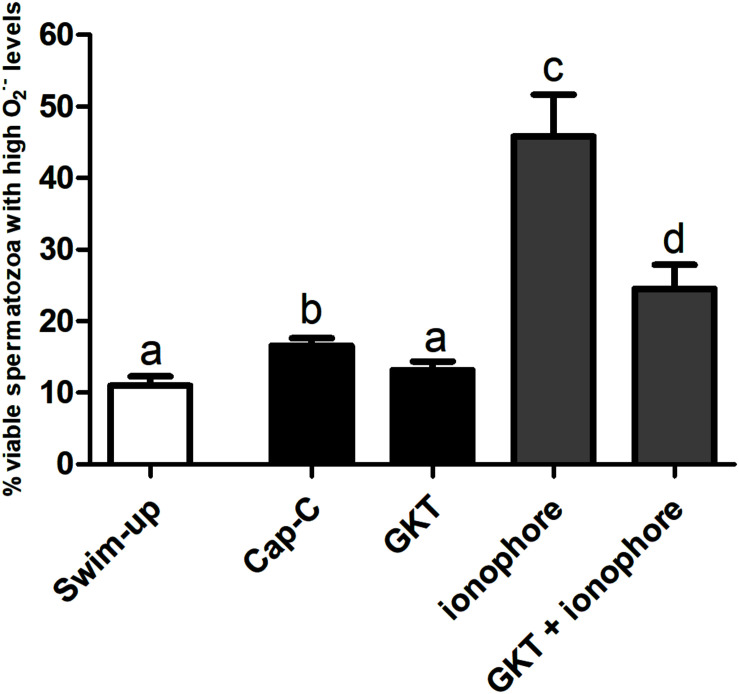
Percentage of live spermatozoa with high superoxide levels (Yo-Pro-1–/E+) assessed by flow cytometry before (swim-up, white bars) and after *in vitro* capacitation with cAMP-elevating agents (Cap-C, black bars) and with GKT136901 (GKT, 1 μM) or/and calcium ionophore A23187 (ionophore, 1 μM, gray bars). Data are shown as mean ± S.E.M. (*n* = 6). Different letters indicate significant differences (*p* < 0.05).

Superoxide intracellular levels (O_2_^⋅–^), were assessed by flow cytometry, and the results are shown in Figure 3. *In vitro* capacitation with cAMP-elevating agents provoked an increase in the percentage of live spermatozoa with high O_2_^⋅–^ levels (Yo-Pro-1-/E+), and the incubation with the NOX5 inhibitor partially limited this increase (*p* < 0.05). Meanwhile, the activation of NOX5 by the calcium ionophore trebled the Yo-Pro-1-/E + population (*p* < 0.0001). When the calcium ionophore was added in the presence of the GKT, there was still an increase of O_2_^⋅–^ (*p* < 0.0001), but to a much lesser extent (24.53% ± 3.35%) than when the ionophore alone was added (45.83% ± 5.83%) (Figure 3).

#### Influence of NOX5 on Capacitation Status During Incubation in Capacitating Conditions

**FIGURE 4:**
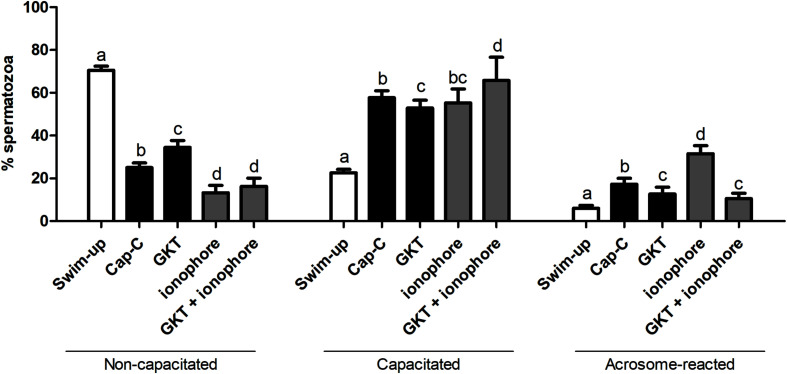
Assessment of capacitation status, evaluated by chlortetracycline (CTC), in ram spermatozoa before (swim-up, white bars) and after *in vitro* capacitation with cAMP-elevating agents (Cap-C, black bars) and with GKT136901 (GKT, 1 μM) or/and calcium ionophore A23187 (ionophore, 1 μM, gray bars). Data of non-capacitated, capacitated, and acrosome-reacted spermatozoa are mean percentages ± SEM (*n* = 6). Different letters within the same group indicate significant differences (*p* < 0.05).

According to the chlortetracycline analysis (CTC), the percentage of capacitated spermatozoa significantly increased after a 3 h incubation under capacitating conditions (Cap-C, 57.71% ± 3.22%) compared to the swim-up samples (22.66% ± 1.5%, *p* < 0.05), as shown in Figure 4. The NOX5 inhibitor partially prevented sperm capacitation (52.85% ± 3.68%, *p* < 0.05). On the other hand, the addition of the calcium ionophore caused a significant percentage of acrosome-reacted spermatozoa (*p* < 0.0001). When GKT was added together with the calcium ionophore, there was a significant reduction in the percentage of acrosome-reacted spermatozoa compared with the same samples in the presence of the ionophore alone (10.50% ± 2.50% vs. 31.50% ± 3.80%, *p* < 0.0001).

#### Influence of NOX5 on Sperm Motility During Incubation in Capacitating Conditions

**FIGURE 5:**
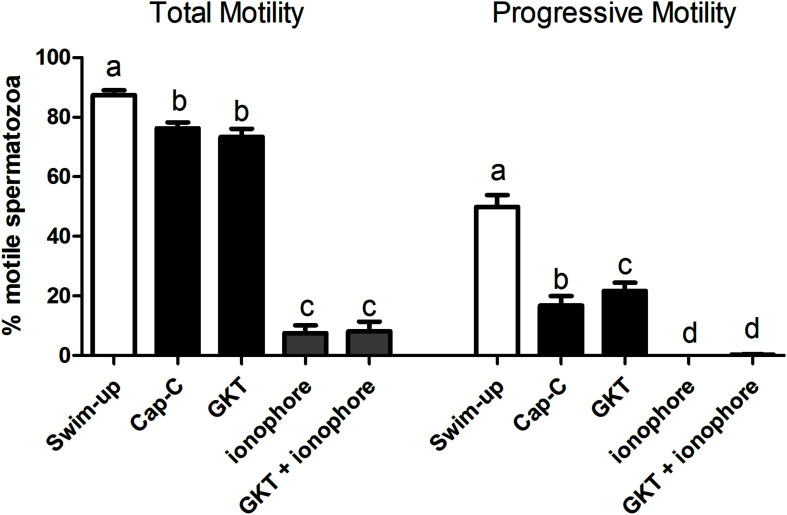
Percentage of total motile (left) and progressive (right) spermatozoa before (swim-up, white bars) and after *in vitro* capacitation with cAMP-elevating agents (Cap-C, black bars) and with GKT136901 (GKT, 1 μM) or/and calcium ionophore A23187 (ionophore, 1 μM, gray bars). Data are shown as mean ± SEM (*n* = 6). Different letters indicate significant differences (*p* < 0.05).

Total and progressive motility significantly decreased after *in vitro* capacitation with cAMP-elevating agents (Figure 5). No significant effects on total motility were found in the presence of GKT, but the NOX5 inhibitor was able to increase progressive motility (15.63% ± 4.45% vs. 22.18% ± 3.77%, *p* < 0.001). However, the addition of the calcium ionophore dramatically compromised total motility (*p* < 0.0001) and the spermatozoa were not able to move progressively at all. The presence of GKT was not able to revert this effect. Viability evaluation revealed that sperm membrane integrity was not compromised in any of the experimental conditions (Supplementary Figure 2).

### Influence of Melatonin on the NOX5 Action on ram Spermatozoa

**FIGURE 6:**
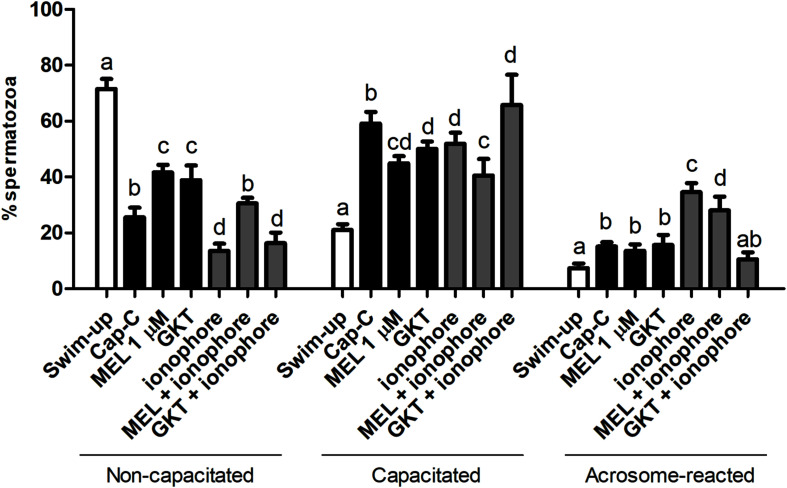
Assessment of capacitation status, evaluated by CTC, in ram spermatozoa before (swim-up, white bars) and after *in vitro* capacitation with cAMP-elevating agents (Cap-C, black bars) and with melatonin (MEL, 1 μM), GKT163901 (GKT, 1 μM), calcium ionophore A23187 (ionophore, 1 μM, gray bars), and a combination of MEL or GKT with ionophore. Data of non-capacitated, capacitated, and acrosome-reacted spermatozoa are mean percentages ± SEM (*n* = 6). Different letters within the same group indicate significant differences (*p* < 0.05).

Once the existence and localization of the NADPH oxidase 5 in ram spermatozoa and its effects on sperm capacitation and functionality were confirmed, we continued further to elucidate whether the action of melatonin on ram sperm capacitation is mediated by its interaction with NOX5. Previously, we checked the effectiveness of the incubation in capacitating conditions and the already described decapacitating action of melatonin at 1 μM concentration on ram spermatozoa (Casao et al., 2010b) by CTC analysis. Melatonin was able to reduce the percentage of capacitated spermatozoa in Cap-C samples (44.87% ± 4.58% vs. 59.13% ± 4.16%, *p* < 0.001, Figure 6) in a similar extent to the NOX5 inhibitor (GKT, 50% ± 2.74%). Moreover, the incubation with melatonin ameliorated the ionophore effects, presenting a lower percentage of capacitated and acrosome-reacted spermatozoa, and more non-capacitated (*p* < 0.01) than in the ionophore samples.

#### Effect of Melatonin on Superoxide Levels

**FIGURE 7:**
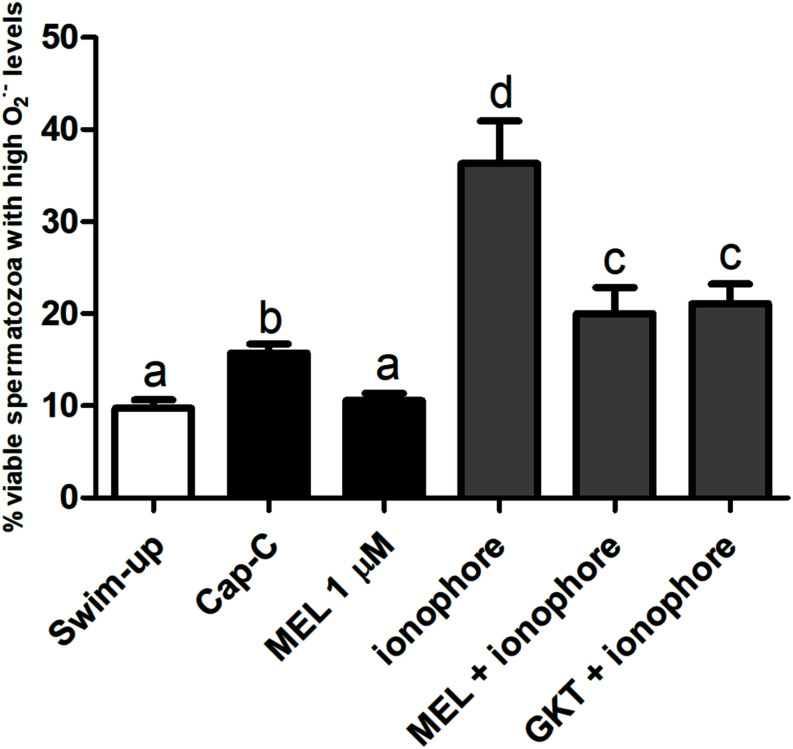
Percentage of live sperm with high superoxide levels (Yo-Pro-1–/E+) assessed by flow cytometry before (swim-up, white bars) and after *in vitro* capacitation with cAMP-elevating agents (Cap-C, black bars) and with melatonin (MEL) 1 μM or/and calcium ionophore A23187 (ionophore, gray bars), plus a GKT136901 with ionophore sample (GKT + ionophore). Data are shown as mean ± SEM (*n* = 6). Different letters indicate significant differences (*p* < 0.05).

Regarding the production of the superoxides, melatonin prevented the superoxide production in live spermatozoa to a significant degree when compared to Cap-C samples (*p* < 0.01, Figure 7), even maintaining the same levels as before the capacitation induction (swim-up samples). The addition of calcium ionophore to the melatonin-preincubated sample did not reach the same superoxide levels as when added alone. Thus, melatonin prevented the superoxide production at the same level as the NOX5 inhibitor GKT136901 did.

#### Effect of Melatonin on NOX5 Levels and Immunolocation in Capacitated Samples

**FIGURE 8:**
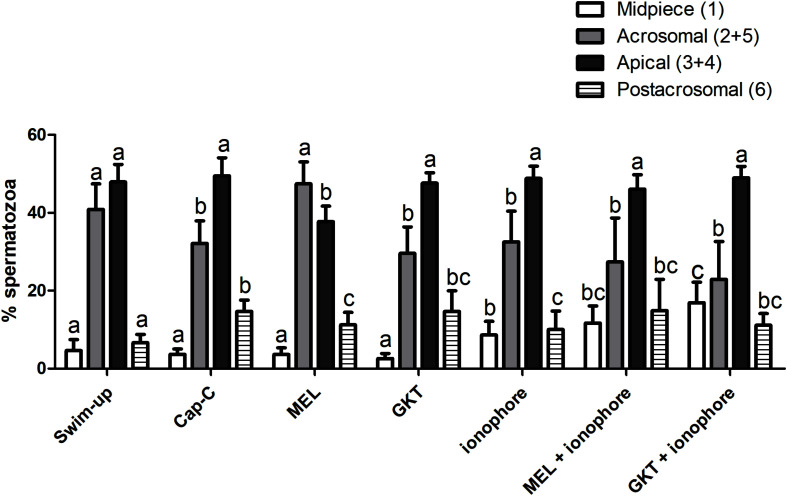
Percentages of the immunotypes for NOX5 in swim-up selected and *in vitro* capacitated ram spermatozoa with cAMP-elevating agents (Cap-C), and with melatonin (MEL) 1 μM, GKT163901 (GKT, 1 μM), calcium ionophore A23187 (ionophore, 1 μM), and a combination of MEL or GKT with ionophore. Immunotypes are represented as follows: (1) plain white bars (2 + 5) plain gray bars (3 + 4) plain black bars, and (6) horizontal striped bars. Results are shown as mean ± SEM (n = 6 for swim-up, Cap-C and Mel; *n* = 3 for GKT, ionophore, MEL + ionophore, GKT + ionophore). Different letters indicate statistical differences between treatments (*p* < 0.05).

In order to better understand the results, and since the postacrosomal labeling was sometimes diffuse, we grouped those immunotypes that labeled the acrosomal region (immunotypes 2 and 5) and the apical edge (immunotypes 3 and 4) regardless of the postacrosomal staining (Figure 8). Before capacitation, in the swim-up selected samples, the primary localization for NOX5 was in the acrosomal region (40.85% ± 6.549417% inmunotypes 2 + 5) and the apical edge (47.9% 4 ± 4.47% immunotypes 3 + 4). The induction of *in vitro* capacitation with cAMP-elevating agents (Cap-C samples) produced a different redistribution of NOX5 (*p* < 0.05), with a lesser proportion of acrosomal immunotypes (2 + 5) and a higher percentage of immunotype 6 (postacrosomal region).

The addition of 1 μM melatonin resulted in the spermatozoa showing different labeling patterns. Thus, melatonin partially prevented the rise in the percentage of immunotype 6 that *in vitro* capacitation caused (*p* < 0.05) and led to an inversion in the proportion of acrosomal (47.41% ± 5.68%) and apical (37.74% ± 3.97%) immunotypes, compared to the Cap-C samples (32.140% ± 5.80% and 49.48% ± 4.63% respectively).

The incubation with GKT during sperm capacitation or the final addition of calcium ionophore to the samples did not provoke significant changes in the percentage of acrosomal and apical immunotypes comparing to the Cap-C samples. However, there was a higher proportion of midpiece labeling in all the samples incubated with ionophore (*p* < 0.05), and the combination of melatonin and GKT with calcium ionophore did not show significant differences compared to the ionophore samples.

**FIGURE 9:**
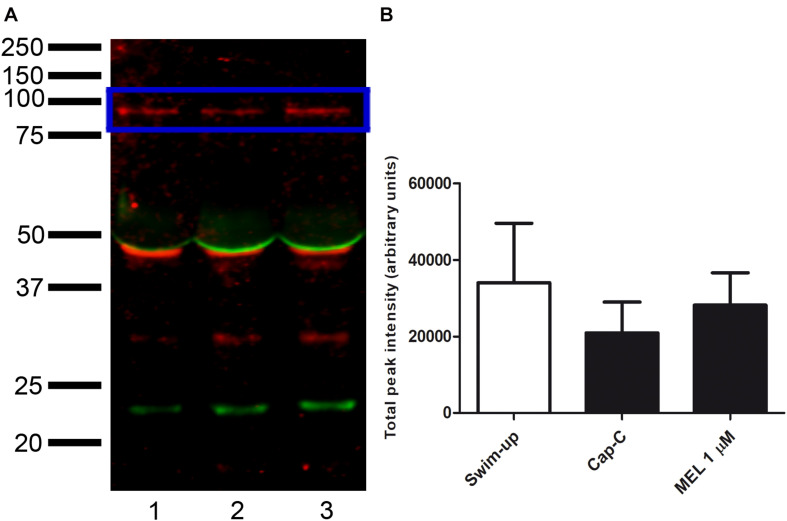
**(A)** Western blot analysis of the presence of NADPH oxidase 5 (blue box) in swim-up selected (lane 1) and incubated 3 h in capacitating conditions with cAMP-elevating agents (Cap-C, lane 2) ram spermatozoa protein extracts. Effect of melatonin 1 μM (MEL 1 μM) in capacitated samples with high cAMP (lane 3). **(B)** Densitometry quantification of NOX5 normalized to α-tubulin (loading control, orange box) (*n* = 6).

Western blot assays were performed to investigate whether the melatonin effects on ram sperm-capacitated samples were mediated by changes in the NOX5 levels (Figure 9). The quantification of NOX5 bands by densitometry, after their normalization with the α-tubulin loading control (∼50 kDa), revealed no significant differences between the samples.

## Discussion

The discovery of NADPH oxidases clarified the source of ROS production in several cell types (Panday et al., 2015). In spermatozoa, the superoxide production was attributed, until several years ago, solely to leukocytes present in semen samples and related to pathological processes (Plante et al., 1994; Whittington et al., 1999). Nowadays, it is commonly assumed that the generation of ROS in spermatozoa would be mediated by autonomous NADPH oxidase activity and that certain levels of ROS would be necessary for some physiological events (reviewed in Bedard and Krause, 2007). NOX5 was discovered in 2001 in human testis, uterus, spleen, and lymph node (Banfi et al., 2001; Cheng et al., 2001). Some years later, it was found in equine (Sabeur and Ball, 2007), human (Musset et al., 2012; Ghani et al., 2013) and canine spermatozoa (Setyawan et al., 2016).

In the present study, we have demonstrated, for the first time, the presence of the NOX5 isoform in ram spermatozoa by Western blot and by indirect immunofluorescence. The 85-kDa protein band detected on the immunoblot matched with the protein detected in equine spermatozoa (Sabeur and Ball, 2007) and similarly with the 75 kDa protein in human spermatozoa (Musset et al., 2012). Likewise, the lower molecular weight protein bands also detected in ram sperm had previously been observed in equine spermatozoa, and the authors noted that they could correspond to proteolytic processes (Sabeur and Ball, 2007).

According to NOX5 localization in ram spermatozoa, we have described six different immunotypes, in contrast with the one or two immunotypes found in human (Musset et al., 2012) and equine (Sabeur and Ball, 2007) spermatozoa, respectively. Thus, NOX5 localization in spermatozoa seems to vary depending on the species. A significant finding of the present work is the evidence, for the first time, that incubation in capacitating conditions triggered a redistribution of NOX5 in ram spermatozoa. These findings suggest that the NOX5 could be involved in ram sperm capacitation, probably by regulating ROS production.

NOX5 product (superoxide anion) increased its levels in samples incubated under capacitating conditions, as reported in human, equine, and boar spermatozoa (de Lamirande and Gagnon, 1995; O’Flaherty et al., 2005; Burnaugh et al., 2007). Activation of NOX5, by the addition of ionophore A23187, dramatically increased superoxide levels in ram spermatozoa, as previously reported in human and equine (Griveau and Le Lannou, 1994; Aitken et al., 1997; Ball et al., 2001; Burnaugh et al., 2007). Inhibition of NOX5 by the addition of the NOX inhibitor (GKT136901) reduced the superoxide generation in samples incubated in capacitating conditions in the presence of cAMP-elevating agents.

We have also demonstrated that NOX5 is involved in ram sperm capacitation, since its inhibition by GKT136901 reduced the percentage of capacitated and acrosome-reacted spermatozoa that had increased during incubation in capacitating conditions. NOX5 activation by ionophore led to a high increment in acrosome-reacted sperm that could be prevented in part by the addition of GKT136901. It is well known that A23187 induces the acrosome reaction in the sperm of many species (Yanagimachi, 1975) and can also immobilize them (Hong et al., 1985; Visconti et al., 1999). In the present study, we observed the same ionophore side effect on motility; however, it did not affect viability, in concordance with the findings of other authors (Suarez et al., 1987). In contrast with different results showing a reduction of sperm motility and viability as a consequence of the inhibition of the NOX5 activity (Ghanbari et al., 2018), we observed an increase in the progressive motility in high cAMP-capacitated samples.

Although other NOX isoforms have been described in spermatozoa from other species (Sabeur and Ball, 2007; Musset et al., 2012; Ghani et al., 2013; Setyawan et al., 2016), on the basis of the results derived from the use of the NOX inhibitor (GKT136901) and the calcium induction, we could postulate that NOX5 is an important source of ROS in ram spermatozoa. This isoform is the only one that has calcium-binding sites, and calcium is crucial for NOX5 activation (reviewed by Touyz et al., 2019). Consequently, the superoxide levels reached after calcium-ionophore addition supported the view that NOX5 is a ROS source in ram spermatozoa. Other authors concluded the same for spermatozoa from other species and affirmed that NOX5 regulates numerous redox-dependent processes in spermatozoa under physiological conditions (Musset et al., 2012; Ghanbari et al., 2018).

**FIGURE 10:**
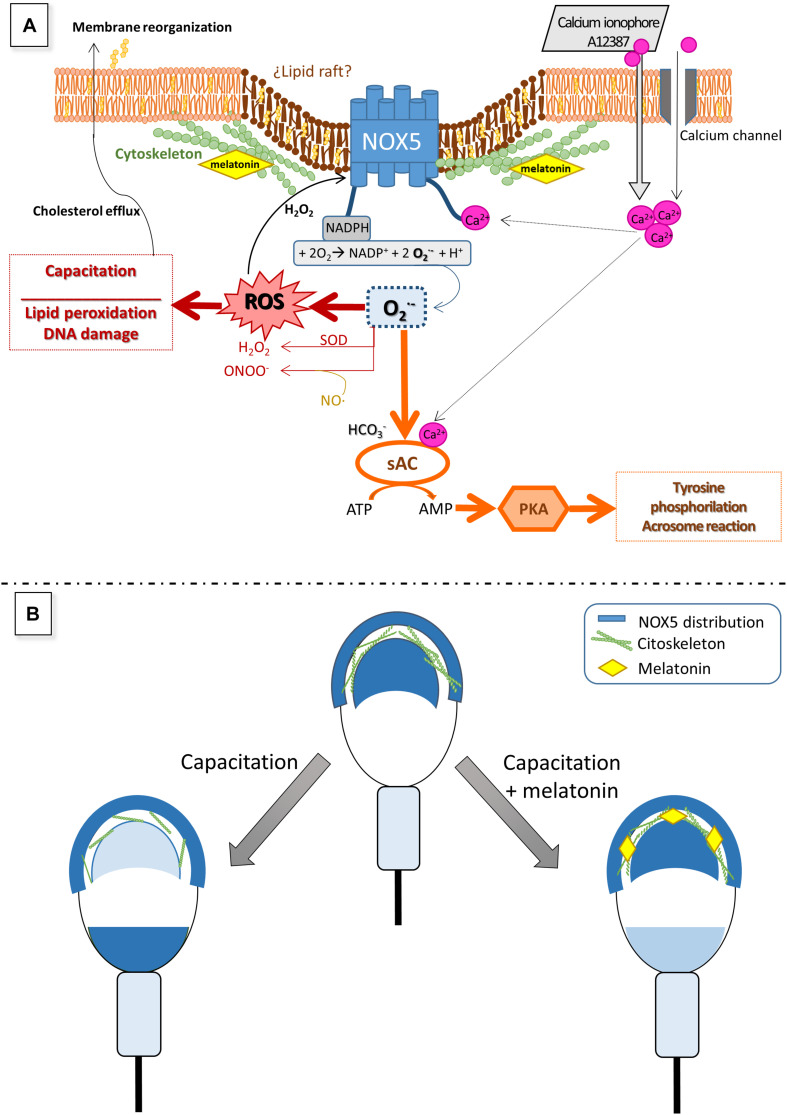
Hypothetical model to explain the role of NOX5 in ram spermatozoa **(A)** and changes in its localization during sperm capacitation **(B)**, as well as the putative effect of melatonin on NOX5 **(A,B)**. Calcium entry from the extracellular media through multiple calcium channels in sperm membrane directly activates NOX5 throughout the N-terminal calcium-binding domains. NOX5 activation generates superoxide which quickly dismutates in H_2_O_2_ and other ROS that take part in numerous signaling pathways, including capacitation processes and NOX5 reactivation. Lewis and Aitken (2001) also proposed that superoxides, together with bicarbonate and calcium, can activate a soluble adenyl cyclase (sAC) which produces cAMP leading to the activation of protein kinase A (PKA). PKA provokes an increase in tyrosine phosphorylation, a crucial event for the ongoing of the capacitation process. The findings of the present work showing that the NOX inhibitor, GKT136901, is able to reduce the rate of ram-capacitated spermatozoa, will support this postulated mechanism. Regarding NOX5 localization, mainly in the acrosome region and the apical edge, we have demonstrated that it changes during ram sperm capacitation. To explain this redistribution we could hypothesize that NOX5 could be embedded in lipid rafts, as it has been demonstrated in somatic cells (Anagnostopoulou et al., 2020). During the capacitation process, the cholesterol efflux from the sperm membrane allows the reorganization of the lipid rafts (van Gestel et al., 2005), giving new protein distributions, and modulating their activity (Kawano et al., 2011). This lipid raft redistribution was demonstrated to be the mechanism by which NOX5 could modulate its activity, in certain somatic cells (Anagnostopoulou et al., 2020). The cytoskeleton plays a fundamental part in the rearrangement of the membrane during sperm capacitation (Brener et al., 2003). Our previous results showed that melatonin modulates ram sperm capacitation and stabilizes the sperm cytoskeleton at micromolar concentrations. Considering these evidences, we postulate that melatonin is able to avoid NOX5 changes in localization associated with sperm capacitation by means of cytoskeleton stabilization, resulting in a lower superoxide production. Panel **(A)** of the figure is based on a model proposed by Aitken and Baker (2020).

Melatonin plays an important role in sperm functionality (Jang et al., 2010; Ortiz et al., 2011), and its antioxidant capacity has been reported in spermatozoa from different species (Succu et al., 2011; Karimfar et al., 2015; Pang et al., 2016; Appiah et al., 2019). It has been demonstrated that, in ram spermatozoa, melatonin is involved in the capacitation process and prevents apoptosis and oxidative stress (Casao et al., 2010b; Gonzalez-Arto et al., 2016; Gimeno-Martos et al., 2019; Pool et al., 2021). In previous works, we showed the capacity of melatonin to prevent ram sperm capacitation at micromolar concentrations (Casao et al., 2010b), in concordance with the present results. This effect appears to be due, at least in part, to its antioxidant capacity, since a melatonin-mediated decrease in general ROS levels in ram (Gimeno-Martos et al., 2019), rabbit (Zhu et al., 2019), bull (Ashrafi et al., 2013), or mouse (Chen et al., 2016) has been described. In contrast, du Plessis et al. (2010) reported no melatonin effect on ROS levels in human spermatozoa, neither did Pool et al. (2021) in frozen-thawed ram spermatozoa, but interestingly, they concluded that melatonin is specifically able to reduce mitochondrial superoxide production. In this work, we have shown that the addition of 1 μM melatonin to the ram sperm samples specifically prevented the increase in superoxide levels provoked by *in vitro* capacitation. This evidence points to a possible action of melatonin through the NOX5 enzyme, an important source of superoxide in spermatozoa. Furthermore, the incubation with melatonin prior to the addition of calcium ionophore limited the superoxide production that the ionophore triggered alone, as did the NOX inhibitor (GKT136901). Moreover, we have shown here that melatonin was able to modify the NOX5 distribution in ram spermatozoa, preventing the changes in localization provoked by *in vitro* capacitation. It is worth mentioning that we have recently reported the effect of melatonin on cytoskeletal remodeling during *in vitro* ram sperm capacitation, concluding that 1 μM melatonin prevents such changes, according to the actin and α-tubulin distribution (Carvajal-Serna et al., 2020). The melatonin action on the cytoskeleton has been also reported in somatic cells (Benítez-King, 2006). Thus, we could postulate that melatonin could affect NOX5 distribution and modulate its capacity to produce superoxides through this stabilizing action on the cytoskeleton, as we proposed in the hypothetical model shown in Figure 10. On the contrary, the incubation with the NOX inhibitor or the calcium ionophore addition did not alter the NOX5 distribution during sperm capacitation, probably because these compounds have a direct modulating action on the enzyme and they could not modify its distribution by themselves. The addition of ionophore after incubation with melatonin avoided its stabilizing effect on cytoskeleton, probably as a consequence of the high increment in intracellular calcium. The fact that NOX5 levels, analyzed by Western blot and quantified by densitometry, did not significantly change after incubation in capacitating conditions, with or without melatonin, may support the previous hypothesis that NOX5 could regulate its action depending on its cell location.

The results from this work have evidenced, for the first time, the presence of NADPH oxidase 5 in ram spermatozoa and the existence of six different immunotypes depending on its location. NOX5 is active and involved in ram sperm capacitation. Finally, melatonin modified the NOX5 location and prevented the oxidative stress deriving from the capacitation process. The findings of this study can be understood as a new insight into the action of melatonin on ram sperm capacitation through NOX5 modulation.

## Data Availability Statement

The datasets generated for this study can be found in the figshare repository doi: 10.6084/m9.figshare.13603226, Supplementary Material can be found in the figshare repository doi: 10.6084/m9.figshare.13606394.

## Ethics Statement

The animal study was reviewed and approved by PI39/17 approved by the University of Zaragoza Ethics Committee for Animal Experiments.

## Author Contributions

RP-P and AC: conceptualization, supervision, and writing—review and editing. SM-J, BP-B, SG-M, and MC-S: methodology. SM-J, BP-B, SG-M, and AC: formal analysis. SM-J, MC-S, BP-B, RP-P, and AC: investigation. SM-J, BP-B, RP-P, and AC: data curation. SM-J: writing—original draft preparation. RP-P: project administration, funding acquisition. All authors have read and agreed to the published version of the manuscript.

## Conflict of Interest

The authors declare that the research was conducted in the absence of any commercial or financial relationships that could be construed as a potential conflict of interest.
