# Supplementary material for: NADPH Oxidase 5 and Melatonin: Involvement in Ram Sperm Capacitation
Source: Front Cell Dev Biol. 2021 May 7;9:655794. doi: 10.3389/fcell.2021.655794 (PMC8138477; doi:10.3389/fcell.2021.655794)
Supplement: Supplementary file 1 [file Data_Sheet_1.PDF]

Supplementary Material

1 Supplementary Figures

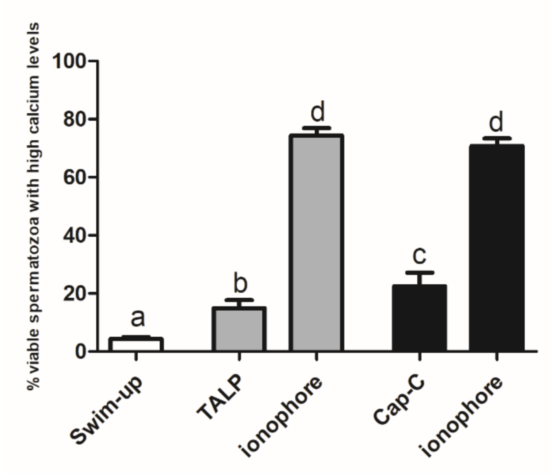

Supplementary Figure 1. Percentage of live spermatozoa with high calcium levels (PI-/Fluo- 4-AM+) assessed by flow cytometry before (swim-up, white bars) and after in vitro capacitation without (TALP, grey bars) and with cAMP-elevating agents (Cap-C, black bars) and with calcium ionophore A23187 (ionophore, 1 µM). Data are shown as mean ± S.E.M. (n = 6). Different letters indicate significant differences ($p < 0.05$).

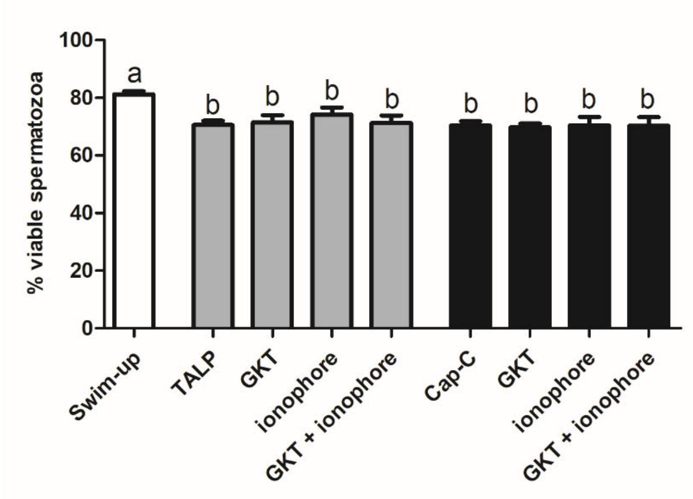

Supplementary Figure 2. Percentage of viable spermatozoa before (swim-up, white bars) and after in vitro capacitation without (TALP, grey bars) and with cAMP-elevating agents (Cap-C, black bars) and with GKT136901 (GKT, 1 µM) or/and calcium ionophore A23187 (ionophore, 1 µM). Data are shown as mean ± S.E.M. (n = 6). Different letters indicate significant differences ($p < 0.05$).

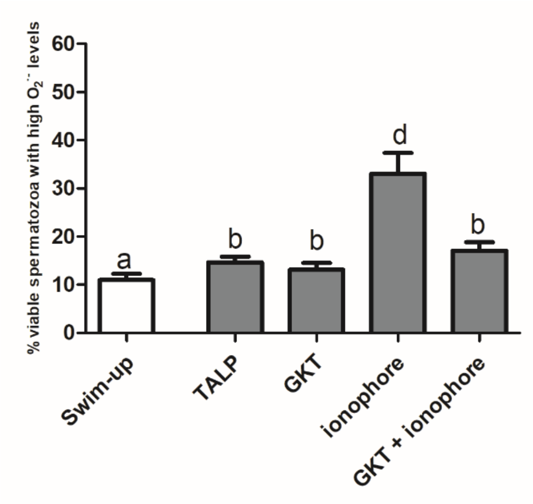

Supplementary Figure 3. Percentage of live spermatozoa with high superoxide levels (Yo-Pro-1-/E+) assessed by flow cytometry before (swim-up, white bars) and after in vitro capacitation without cAMP-elevating agents (TALP, grey bars) and with GKT136901 (GKT, 1 µM) or/and calcium ionophore A23187 (ionophore, 1 µM). Data are shown as mean ± S.E.M. (n = 6). Different letters indicate significant differences ($p < 0.05$).

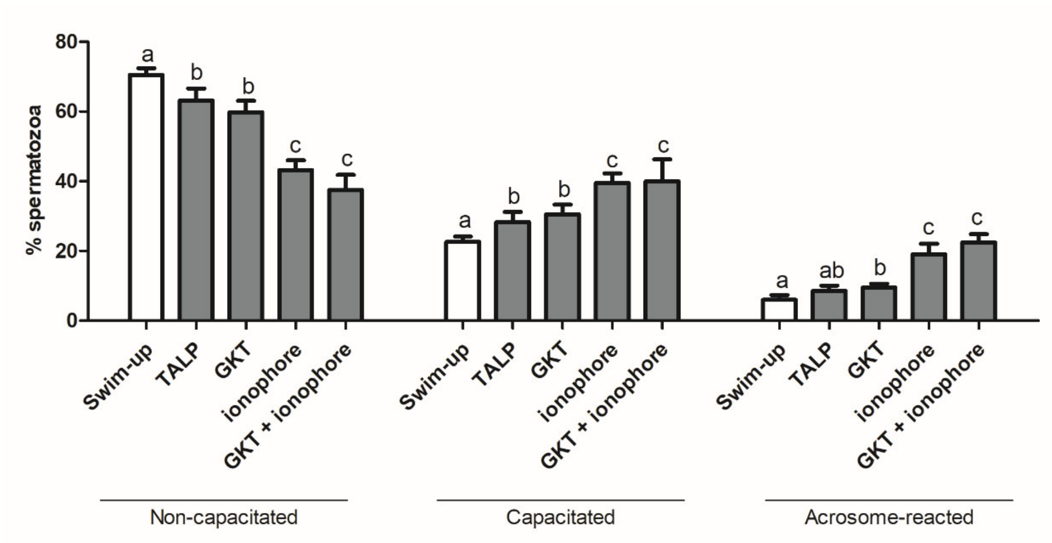

Supplementary Figure 4. Assessment of capacitation status, evaluated by chlortetracycline (CTC), in ram spermatozoa before (swim-up, white bars) and after in vitro capacitation without cAMP-elevating agents (TALP, grey bars) and with GKT136901 (GKT, 1 µM) or/and calcium ionophore A23187 (ionophore, 1 µM). Data of non-capacitated, capacitated and acrosome-reacted spermatozoa are mean percentages ± S.E.M (n = 6). Different letters within the same group indicate significant differences ($p < 0.05$).

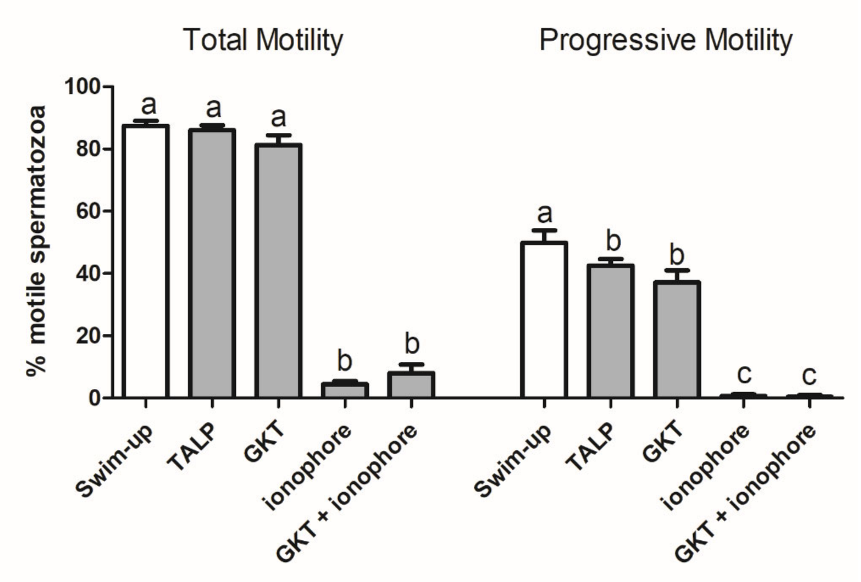

Supplementary Figure 5. Percentage of total motile (left) and progressive (right) spermatozoa before (swim-up, white bars) and after in vitro capacitation without cAMP-elevating agents (TALP, grey bars) and with GKT136901 (GKT, 1 μM) or/and calcium ionophore A23187 (ionophore, 1 μM). Data are shown as mean ± S.E.M. (n = 6). Different letters indicate significant differences ($p < 0.05$).
